# Editorial: News in Graves’ orbitopathy: patients management and treatments

**DOI:** 10.3389/fendo.2023.1270467

**Published:** 2023-08-18

**Authors:** Giulia Lanzolla, Elena Sabini

**Affiliations:** ^1^ Department of Clinical and Experimental Medicine, Endocrinology Unit II, University of Pisa and University Hospital of Pisa, Pisa, Italy; ^2^ Department of Orthopaedic Surgery, University of Pennsylvania, Philadelphia, PA, United States

**Keywords:** Graves’ disease (GD), thyroid autoimmunity, thyroid eye disease (TED), Graves’ orbitopathy, thyroid, thyroid disease

Graves’ orbitopathy (GO) is an autoimmune disease observed in ~30% of patients with Graves’ disease (GD) (1). Despite advancements in understanding, the current treatment of GO is not satisfactory. The ultimate pathogenetic mechanism is still being defined, and new risk factors, diagnostic methods, and treatment procedures have been proposed. This Research Topic consists of 11 insightful contributions that offer a timely overview of GO, from terminology to treatment novelties.

Currently, there is no widely accepted name for GO. Wagner et al. proposed thyroid eye disease (TED) as the preferred term since it is likely to give patients a better understanding of the disease. The authors discussed the appropriateness of “GO”, which includes the eponymous “Graves” even though a small percentage of patients are not affected by GD. Despite TED gaining popularity among ophthalmologists, GO remains the term most used by endocrinologists. Interestingly, publications by multidisciplinary author teams showed an increase in using TED from 2000 to 2020, although GO remains the dominant term. While the change proposed by Wagner is intriguing, “TED” does not fully encompass the disease, since it is an “orbitopathy” that affects all structures within the orbit rather than exclusively the eye. Overall, a universally accepted terminology is necessary, thereby minimizing confusion and dichotomy among various specialists.

Given the knowledge that GO is due to a complex interplay between innate immunity, humoral immunity, and inflammatory response, several factors affecting immune tolerance and inflammation may have triggering or protective and therapeutic roles (1, 2). In a comprehensive retrospective study, Oeverhaus et al. confirmed that age, male sex, smoking, GD, and radioiodine are important risk factors for the development of severe stages of GO. Park et al. proposed the machine learning system eXtreme Gradient Boosting to predict the response to intravenous glucocorticoids (ivGCs) in GO patients. TSH, thyroid-stimulating immunoglobulins (TSI), and low-density lipoprotein cholesterol (LDL-C) were the features mostly influencing responsiveness. LDL-C had the greatest impact on the AI model, confirming it as a risk factor that can influence GO course and ivGCs response. Given the sample size and retrospective design, further, larger prospective studies are needed to validate this predictive system.

One of the most dreaded complications of GO is the impairment of visual function, caused by optic nerve compression at the orbital apex. Retinal and choroidal microvascular density have been proposed to evaluate the early stage of visual impairment in patients with GO (3, 4). Zeng et al. performed a cross-sectional study to assess macular vessel density (VD) in GO patients with chorioretinal folds (CRFs) with and without optic disc edema. They found a significant decrease in macular VD in GO patients with CRFs, which correlated with visual dysfunction, offering a new possible index to be considered in GO patients’ management. Retinal perfusion is another parameter that can be investigated in patients with GO and visual field defects. Patients with sight-threatening GO may experience vascular insufficiency of retinal perfusion due to continuous mechanical compression and optic nerve stretching (5). Ye et al. showed that elevated pulse pressure correlate with reduced retinal peripapillary perfusion in GO patients, leading to visual field defects. These results suggest that vascular insufficiency may contribute to visual impairment in patients with GO by reducing retinal perfusion.

Immunosuppressive treatment is reserved for moderate-to-severe active GO, making assessment of GO activity a key element in determining the most appropriate treatment (2). Tissue inflammation is commonly evaluated using the clinical activity score (CAS) (2, 6). Even though it is a standardized and useful tool, CAS carries several limitations. Given the importance of GO activity in driving patient management, a more comprehensive score including several assessment factors, is needed. Since magnetic resonance imaging (MRI) provides information on orbital tissue expansion and distribution (2, 7, 8), Song et al. reviewed the potential application of MRI in quantifying GO activity. They confirmed that T2 relaxation time can be used to quantify GO activity and may aid in predicting the response to anti-inflammatory treatment. In the attempt to find new useful elements in identifying patients with active GO, Olejarz et al. proposed immunoglobulin G4 (IgG4) as a marker of GO activity. The role of IgG4 in thyroid disease has been investigated and high IgG4 levels have been reported in GD and GO, although their exact role and significance remain unclear (9–12). Olejarz et al. proposed a prospective observational study in which 60 patients with GO were divided into a high IgG4 group (>135 mg/dL) and a normal IgG4 group (<135 mg/dL). The high IgG4 group showed higher prevalence of active GO defined by MRI and higher TRAb titers compared to the normal IgG4 group. However, no significant difference in CAS was observed between the two groups. Further larger studies are needed to clarify the potential significance of high levels of IgG4 in GO before considering it a marker of activity.

Due to the lack of safe and well-tolerated treatments that guarantee a complete and satisfactory response, GO patients represent a challenge for endocrinologists and ophthalmologists. The targeted therapy with monoclonal antibodies is one of the most promising alternatives for patients with moderate-to-severe active GO (13–16). However, the safety profile and best-recommended dose are not yet fully defined. Wang et al. investigated the long-term (up to 224 weeks) efficacy and safety of using low doses (125 mg/m^2^ weekly for 4 weeks) of rituximab, a monoclonal antibody that targets CD20 on the surface of pre-B and mature B-lymphocytes, to treat patients with moderate-to-severe active GO. A significant decrease in CAS, exophthalmos, and thickness of extraocular muscles was observed, with no major adverse events, suggesting that low doses of rituximab may be considered for GO patients. Hu et al. in a meta-analysis including 12 trials showed that the IL-6 receptor inhibitor tocilizumab was likely the best treatment for moderate-to-severe GO in terms of indirect contrast response, followed by teprotumumab (IGF-1 receptor blocking monoclonal antibody) and rituximab. Moreover, tocilizumab had the best result in reducing proptosis and a higher safety profile. These data are based on an indirect comparison and need to be confirmed by head-to-head trials. The optimal dose, safety, and long-term efficacy of monoclonal antibodies remain to be established, and this may change the treatment paradigm for GO in the future. Bottom line, monoclonal antibodies represent a new therapeutic area in GO and further randomized controlled studies with an appropriate sample size and study design are needed to confirm the promising results reported by Wang and Hu.

Despite medical progress and the discovery of new target therapies for patients with moderate-to-severe active GO, patients with inactive chronic disease benefit from orbital decompression, squint, and palpebral surgery (2). Savino et al. in a retrospective observational study including 29 patients with strabismus and diplopia compared the long-term effects of two types of eye surgery: bilateral medial recti recession or unilateral inferior rectus recession. Bilateral medial rectus recession leads to significant improvements in the deviation angle and diplopia, with stable under-correction over time, while inferior rectus recession leads to more unstable outcomes. Moreover, the latest advances in GO surgical procedures are reported by Baeg et al. to provide updated insights on the most appropriate choices.

This Research Topic offers encouraging updates on patient management, including advances in both the diagnosis and treatment of GO. The need for standardized terminology and improved disease activity assessment scores are interesting topics that can be investigated in future studies, as well as the possibility of using artificial intelligence systems to predict treatment response rates.

## Author contributions

GL: Conceptualization, Writing – original draft, Writing – review & editing. ES: Writing – review & editing.
